# Real-World Evidence of Systemic Therapy Sequencing on Overall Survival for Patients with Metastatic BRAF-Mutated Cutaneous Melanoma

**DOI:** 10.3390/curroncol29030126

**Published:** 2022-03-01

**Authors:** Adi Kartolo, Jasna Deluce, Wilma M. Hopman, Linda Liu, Tara Baetz, Scott Ernst, John G. Lenehan

**Affiliations:** 1 Department of Oncology, Queen’s University, Kingston, ON K7L 2V7, Canada; 12bak@queensu.ca (A.K.); tara.baetz@kingstonhsc.ca (T.B.); 2 Department of Oncology, University of Western Ontario, London, ON N6A 5W9, Canada; jasna.deluce@lhsc.on.ca (J.D.); scott.ernst@lhsc.on.ca (S.E.); 3 Department of Public Health Sciences, Queen’s University, Kingston, ON K7L 2V7, Canada; wilma.hopman@kingstonhsc.ca; 4 Pulse Infoframe, London, ON N5X 4E7, Canada; lliu@pulseinfoframe.com

**Keywords:** BRAF mutant, melanoma, targeted therapy, immunotherapy, therapy sequencing

## Abstract

Aim: To evaluate optimal systemic therapy sequencing (first-line targeted therapy (1L-TT) vs. first-line immunotherapy (1L-IO)) in patients with BRAF-mutated metastatic melanoma. Methods: Nation-wide prospective data of patients with newly diagnosed BRAF-mutated metastatic melanoma were retrieved from the Canadian Melanoma Research Network. Results: Our study included 79 and 107 patients in the 1L-IO and 1L-TT groups, respectively. There were more patients with ECOG 0–1 (91% vs. 72%, *p* = 0.023) in the 1L-IO group compared to the 1L-TT group. Multivariable Cox analysis suggested no OS differences between the two groups (HR 0.838, 95%CI 0.502–1.400, *p* = 0.500). However, patients who received 1L-TT then 2L-IO had the longest OS compared to 1L-IO without 2L therapy, 1L-IO then 2L-TT, and 1L-TT without 2L therapy (38.3 vs. 32.2 vs. 16.9 vs. 6.3 months, *p* < 0.001). For patients who received 2L therapy, those who received 2L-IO had a trend towards OS improvement compared with the 2L-TT group (21.7 vs. 8.9 months, *p* = 0.053). Conclusions: Our nation-wide prospective study failed to establish any optimal systemic therapy sequencing in advanced BRAF-mutant melanoma patients. Nevertheless, we provided evidence that immunotherapy has durable efficacy in advanced BRAF-mutant melanoma patients, regardless of treatment line, and that Canadian medical oncologists were selecting the appropriate treatment sequences in a real-world setting, based on patients’ clinical and tumour characteristics.

## 1. Introduction

It is well-established that effective systemic therapy options for advanced cutaneous melanoma have dramatically improved since the introduction of immunotherapy and, in the case of BRAF V600E/K-mutated melanoma, targeted therapy. For patients that have melanoma with an activating BRAF mutation, both treatment modalities are available, and typically the choice of which therapy is used first is at the discretion of the treating oncologist. Currently, there are limited data to guide selection of a first-line treatment. Guidelines from leading organizations offer little in the way of direction in this situation [1,2,3]. More recently, the 2019 ESMO Clinical Practice Guidelines suggested first-line therapy decisions need to be individualized according to patients’ clinical status, comorbidities, treatment goals, and personal preferences, although immunotherapy should still be preferred as first-line therapy for its durable disease control even after treatment discontinuation [3].

When examining the randomized phase III data from all therapies, targeted therapy has a superior progression free survival (PFS) and overall survival (OS) compared to immunotherapy within the first 12 months; however, the duration of response is superior with immunotherapy, as is the OS beyond the first year [4,5,6,7,8]. This observation appears to be true in both first- and second-line settings [9]. Clinical trial data have definitely shown that combined BRAF- and MEK-inhibitors are superior to a BRAF-inhibitor alone. Such direct comparison data are not available for anti-PD-1 therapy compared with anti-PD-1 therapy combined with anti-CTLA-4, although data suggests it may be modestly superior, albeit with increased toxicity [4,5,6,7,8]. In the case of a very aggressive presentation of the disease, targeted therapy is often preferred as a first-line treatment because it has both a superior time to response and response rate [5,7]. In the hope to improve survival outcomes, combined targeted therapy and anti-PD-1 therapy have also been examined previously, but failed to demonstrate overall survival benefit, in addition to increased toxicity profiles [10,11,12]. Therefore, while targeted therapy or immunotherapy are both appropriate first-line options, the question remains as to their sequencing optimizations.

A 2020 review published by Schummer et al. examined data from the phase III CheckMate 067 trial and found that treatment naïve patients with a BRAF mutation, compared to without, had a better OS and PFS with single-agent Nivolumab, and a better OS for the combination of Ipilimumab and Nivolumab [13]. They concluded that combination immunotherapy followed by combination targeted therapy in BRAF-mutated patients may be the most effective strategy; however, this was based only on data from a single trial and may not be applicable to real-world patient populations. Currently, systemic therapy sequencing evidence has come from several retrospective assessments of available clinical trial data [9], although head-to-head comparison trials assessing the sequencing of targeted and immunotherapies are ongoing [14]. Additional evidence in support of sequencing these therapies comes from the real-world data available from electronic health records and single-institution case series. To date, the published data from such analyses are predominantly retrospective assessments [15,16,17,18,19]. In general, most studies included small numbers of patients and although some included patients from multiple centres, many were limited to a single-centre dataset. Some of the more recent studies included only patients treated with single-agent anti-PD-1 therapy and not those treated with combination immunotherapy, which limits the application to real-world patients [16,17]. Regardless, the results in general seem to favour the use of immunotherapy as a first-line therapy, followed by second-line targeted therapy in the context of each study’s limitations.

The prospectively collected data from the Canadian Melanoma Research Network (CMRN) includes patients with BRAF-mutated melanoma who were initiated on either immunotherapy or targeted therapy for first-line treatment, followed by the other for second-line treatment. It is expected that the analysis of a large number of patients from several centres will have statistical power to provide observational evidence for choosing a particular first-line therapy and to highlight factors that may guide oncologists in making the best choice for each individual patient.

## 2. Methodology

Patient data were collected prospectively for BRAF-mutated metastatic melanoma patients with at least 1-year follow-up between 2015 and 2019 from the CMRN database involving academic cancer centres across Canada, including the Cancer Centre of Southeastern Ontario, London Health Sciences Centre, Credit Valley Hospital, McGill University Health Centre, Lakeridge Health, Vitalite Health Network, Ottawa Hospital Research Institute, Princess Margaret Hospital, Sunnybrook Hospital, and Tom Baker Cancer Centre. We included all patients with histologically confirmed unresectable locally advanced or metastatic cutaneous melanoma with V600E/K or other targetable subtypes who received at least one cycle of first-line, palliative-intent immunotherapy-based regimen or BRAF ± MEK targeted therapy. We excluded patients who (1) were treated with MEK-inhibitors alone, (2) received first-line single-agent CTLA-4 inhibitors alone, and (3) received sequential lines of therapy of the same mechanism of action.

We retrieved the following information: age at diagnosis, gender, Eastern Cooperative Oncology Group (ECOG) performance status prior to first-line therapy, cancer stage at initiation of first-line treatment, lactate dehydrogenase (LDH) prior to first-line therapy, BRAF-mutation subtypes, number of metastatic sites prior to first-line therapy, presence of brain metastases prior to first-line therapy, the use of palliative radiation therapy (RT), the use of palliative surgery, first-line systemic therapy type/initiation date/end date, second-line systemic therapy type/initiation date/end date, and reason for the switch to second-line treatment.

We categorized patients into 2 groups according to treatment sequences. The first-line targeted treatment (1L-TT) group included patients who received at least one cycle of first-line BRAF ± MEK inhibitors, with or without subsequent PD-1 ± CTLA-4 inhibitors, and vice versa for the first-line immunotherapy (1L-IO) group.

Our study outcome included overall survival (OS), which was defined by the time of first-line treatment to time of death of any cause. Our secondary study outcomes included time from first-line treatment to time of second-line treatment, time from second-line treatment to time of treatment permanent discontinuation, as well as time from second-line treatment to time of death of any cause. Patients were censored if the events of interest had not been reached at data cut-off. To account for the impact of rare non-V600E/K BRAF mutation on study outcomes, we conducted separate survival analyses with the exclusion of such patients.

### Statistical Analysis

All statistical analyses were conducted in IBM SPSS Statistics version 26. We conducted descriptive and univariate analyses via Fisher’s exact test or Chi-Square test (categorical data), and used Mann–Whitney U and Kruskal–Wallis tests (continuous data) to provide an overview of the baseline population characteristics and the relationship with treatment sequence. Kaplan–Meir curves were generated to examine OS. To assess for potential confounders, we used the multivariable Cox proportional hazard regression model to calculate hazard ratio (HR) and its 95% confidence interval (CI) for OS. We set *p* < 0.05 to define statistically significant outcomes. No adjustments were made for multiple comparisons, so inferences from this data should be carefully considered.

## 3. Results

Our study included a total of 186 patients (Table 1). Seventy-nine patients were in the 1L-IO group, and 107 patients were in the 1L-TT group. Within the 1L-IO group, 56/79 (71%) patients received 1L-single ICI, whereas 23/79 patients (29%) received 1L-dual ICI. The majority of 1L-TT (93%) received combined first-line BRAF and MEK inhibitors, rather than BRAF inhibitor monotherapy. Univariate analyses suggested there were more patients with ECOG 0–1 (91% vs. 72%, *p* = 0.023) in the 1L-IO group compared to 1L-TT group. There were no other imbalances between the two groups in terms of age (*p* = 0.374), gender (*p* = 0.530), LDH (*p* = 0.739), cancer stage (*p* = 1.000), number of metastatic sites (*p* = 0.184), baseline brain metastasis (*p* = 0.397), BRAF-mutation subtypes (*p* = 0.326), received palliative RT (*p* = 1.000), or received palliative surgery (*p* = 0.532).

Table 2 reported the sequencing patterns of systemic therapy based on their respective 1L regimen. There were no significant differences between 1L-IO and 1L-TT groups in receiving 2L therapy (44% vs. 52%, *p* = 0.302). However, the reasons for 1L therapy discontinuation differed between 1L-IO and 1L-TT groups (progression 37% vs. 47%, toxicity 21% vs. 12%). Twenty-three percent of 1L-IO were able to complete their first-line treatment.

More patients died before receiving second-line treatment in the 1L-TT group than 1L-IO group (74% vs. 44%, *p* = 0.015). For patients in the 1L-TT group who received 2L therapy, the majority (73%) received anti-PD1 rather than dual anti-PD1 + anti-CTLA4 regimen (27%). Of the 1L-IO group who received 2L therapy, all of them had combined BRAF and MEK inhibitors. There were also no significant differences between the two groups in terms of time on 1L therapy (*p* = 0.586), time on 2L therapy (*p* = 07918), time from 1L therapy initiation date to 2L therapy initiation date (*p* = 0.338), or time from 2L therapy initiation to death/last follow up (*p* = 0.245).

**Figure 1:**
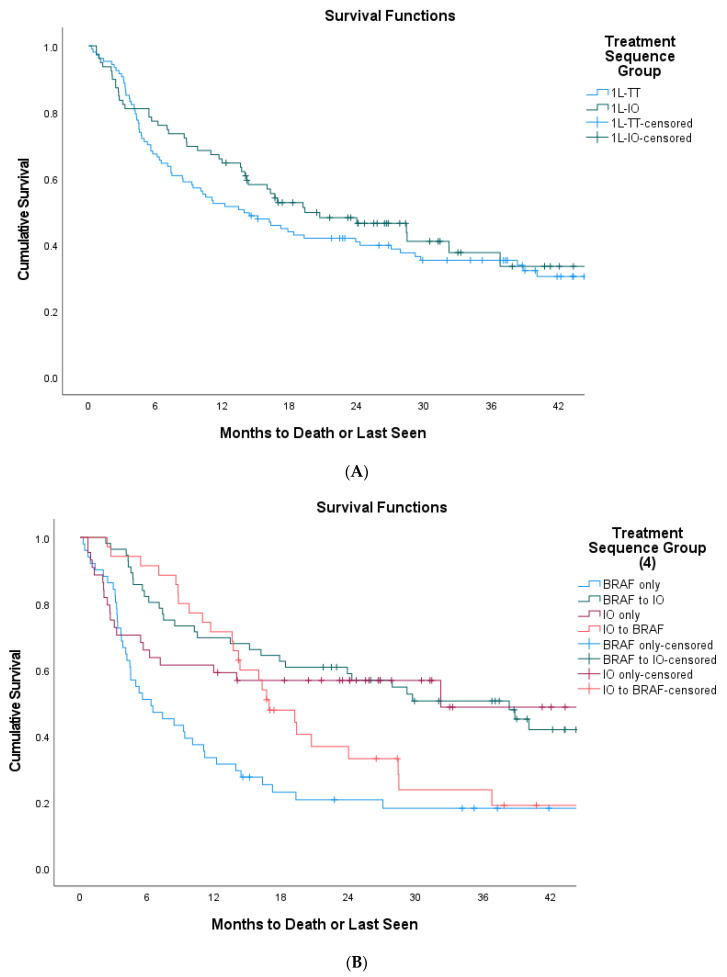

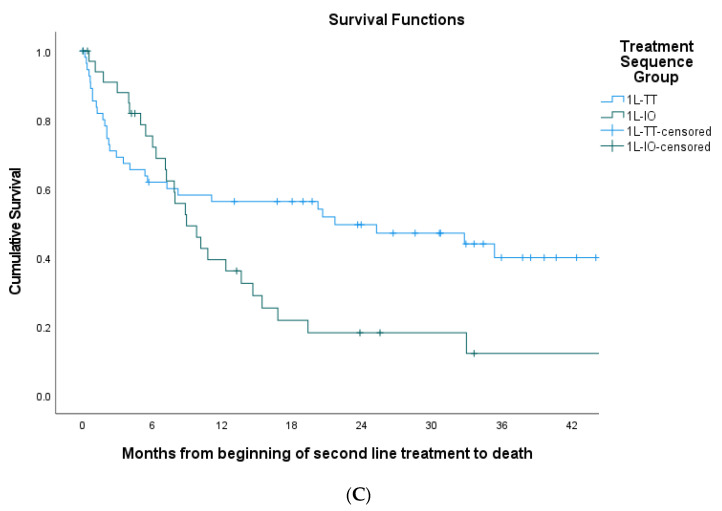
Survival outcomes of BRAF V600-mutant population (*n* = 186). (**A**) Overall survival of BRAF-mutant melanoma patients as per 1L-IO vs. 1L-TT Group (19.3 vs. 13.9 months, *p* = 0.459); (**B**) Overall Survival of BRAF-Mutant Melanoma Patients as per ‘1L-IO only’ vs. ‘1L-TT to 2L-IO’ vs. ‘1L-IO to 2L-TT’ vs. ‘1L-TT only’ (32.2 vs. 38.34 vs. 16.9 vs. 6.3, *p* < 0.001); (**C**) Survival from second-line treatment to time of death/last follow up in BRAF-mutant melanoma patients as per 1L-IO vs. 1L-TT group (8.9 vs. 21.7 months, *p* = 0.053).

There were no statistically significant differences in OS between the two treatment groups (1L-IO 19.3 vs. 1L-TT 13.9 months, *p* = 0.459) (Figure 1A). Upon further stratification of the treatment sequencing patterns, patients who received 1L-TT then 2L-IO had the longest OS compared to 1L-IO without 2L therapy, 1L-IO then 2L-TT, and 1L-TT without 2L therapy (38.3 vs. 32.2 vs. 16.9 vs. 6.3 months, *p* < 0.001) (Figure 1B). For patients who received 2L therapy, those who received 2L-IO had a trend towards OS improvement compared with the 2L-TT group (21.7 vs. 8.9 months, *p* = 0.053) (Figure 1C).

Therapy sequence lost its clinically meaningful OS improvement when potential confounders were accounted for in our multivariable Cox analysis (HR 0.838, 95%CI 0.502–1.400, *p* = 0.500). Instead, number of metastatic sites > 2 (HR 2.195, 95%CI 1.302–3.699, *p* = 0.003), baseline brain metastasis (HR 1.833, 95%CI 1.073–3.130, *p* = 0.027), and baseline ECOG ≥ 2 (HR 3.957, 95%CI 2.226–7.034, *p* < 0.001) were significantly associated with worse OS in BRAF-mutant melanoma patients (Table 3).

**Figure 2:**
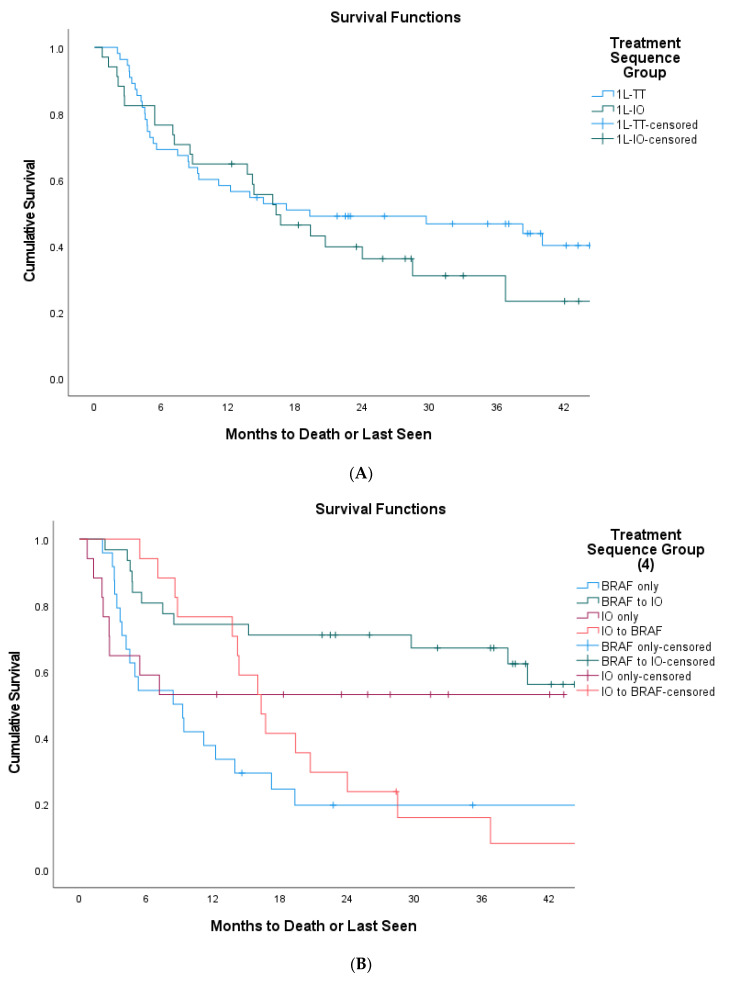

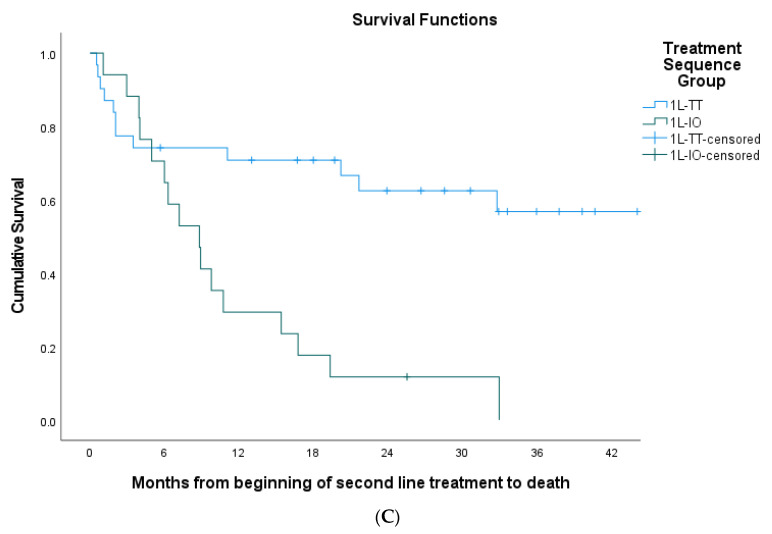
Sensitivity analyses survival outcomes excluding BRAF V600D and unknown subtypes (*n* = 89). (**A**) Overall survival of 1L-IO vs. 1L-TT Group in BRAF V600E/K-mutant population only (16.3 vs. 19.3 months, *p* = 0.230); (**B**) overall survival of ‘1L-IO only’ vs. ‘1L-TT to 2L-IO’ vs. ‘1L-IO to 2L-TT’ vs. ‘1L-TT only’ in BRAF V600E/K-mutant population only (NR vs. NR vs. 16.3 vs. 8.4 months, *p* < 0.001); (**C**) survival from second-line treatment to time of death/last follow up as per 1L-IO vs. 1L-TT group only’ in BRAF V600E/K-mutant population only (8.8 vs. NR, *p* < 0.001).

Upon excluding patients with BRAF V600D and unknown subtype mutations, there were no significant OS differences between 1L-IO and 1L-TT groups (16.3 vs. 19.3 months, *p* = 0.230). However, patients who received 1L-TT then 2L-IO still had the best OS compared to 1L-IO only, followed by 1L-IO then 2L-TT, and 1L-TT only (NR vs. NR vs. 16.3 vs. 8.4 months, *p* < 0.001). Survival from 2L therapy and multivariable Cox analysis showed similar results with or without the exclusion of BRAF V600D patients (Figure 2A–C and Table 3).

## 4. Discussion

We reported a prospective multi-centre study to explore systemic therapy sequencing and its impact on OS in patients with metastatic BRAF-mutant cutaneous melanoma. Overall, there were no statistically significant differences in systemic therapy sequencing patterns in our study. Rather, survival outcomes of BRAF-mutant melanoma patients were impacted by the number of metastatic sites, presence of baseline brain metastasis, and poor baseline functional status.

Compared to other real-world studies, Moser et al. [15] suggested a 34% and 49% mortality risk reduction in patients with BRAF-mutant advanced melanoma who received first-line dual IO versus BRAF + MEK inhibitor and first-line single-agent anti-PD1 versus BRAF + MEK inhibitor, respectively. Pavlick et al. [18] also reported similar findings with a 32% mortality risk reduction favouring first-line dual IO over BRAK + MEK inhibitors in patients with BRAF-mutant advanced melanoma. Both author groups utilized the same Flatiron health database, and it was unclear if the data captured were within the same time frame [15,19]. Taking these into account, the differences in our study compared to others were likely due to the relatively smaller proportion of patients receiving 1L-dual ICI, as most patients in this study were treated before dual-IO became widely available in Canada. It is expected that the benefit of 1L-IO may be similar or become more apparent than 1L-TT when more BRAF-mutant patients receive dual-IO rather than single-IO, as shown in Checkmate 067 [8]. An updated analysis in the future, when more dual ICI is incorporated into the database, would be important to provide additional evidence in this important field.

Nevertheless, our study provided several important insights to the melanoma community. First, our study suggested patients were able to achieve durable responses from IO, even in the second-line setting. In fact, post-2L survival was significantly higher in the 1L-TT group who received 2L-IO when compared to the 1L-TT group who received 2L-TT. Second, we provided evidence that Canadian medical oncologists were likely choosing the optimal first-line systemic therapy based on patients’ clinical and tumour characteristics. Within the limitations of cross-trial comparisons, TT was known to have higher response rate and faster time to response than IO in advanced melanoma management [5,7]. This is also evident in other cancer sites—such as lung cancer [20]—whereby chemotherapy has a better survival benefit than IO at the earlier time in the Kaplan–Meier survival curve and would subsequently change to favouring immunotherapy afterwards. In fact, this phenomenon was eliminated when chemotherapy was given concurrently with immunotherapy as first-line therapy in an effort to increase tumour neoantigens [21,22]. A similar approach of combining TT with IO was evaluated in advanced melanoma patients but has failed to gain traction thus far, mainly due to significant grade 3–4 therapy-related toxicities (39–73%) [23,24,25,26,27,28].

A preliminary result of the SECOMBIT trial, presented in ESMO 2021, demonstrated that 1L-dual ICI and sequential ‘sandwich’ therapy (BRAF and MEK inhibitors for 8 weeks then dual ICI) had better 2-year and 3-year overall survival trends compared to 1L-TT (2-year OS: 73% vs. 69% vs. 65%; 3-year OS: 62% vs. 60% vs. 54%). Along with our study findings, we supported the merit in considering 1L-IO over 1L-TT, in the absence of requiring rapid responses, given that 1L-IO were more likely to complete their treatment and less likely to require 2L therapy due to progression. In patients with a more extensive tumour burden, our study demonstrated that 1L-TT may be considered over 1L-IO. However, our study did not incorporate the ‘sandwich’ sequential pattern. More matured data from SECOMBIT would be required to elucidate any OS benefit in either therapy sequencing pattern [14].

It is difficult to ascertain whether rare but actionable BRAF-mutation subtypes have any impact on systemic therapy sequencing and survival outcomes. This is because our study had only 5 (3%) patients with rare actionable BRAF-mutant patients, although this was in keeping with the prevalence rate of previous studies [29,30], and 92 (49%) patients with unknown BRAF subtypes. Future studies in evaluating optimal treatment sequencing in rare, non-classical BRAF-mutant melanoma patients would be important, especially to identify if TT is less effective in rare BRAF mutations compared to classical BRAF V600E/K mutations [31].

Our study had several limitations which we will address. Firstly, our study was not able to account for the impact of previously received adjuvant IO or TT on subsequent systemic therapy sequencing. Future studies would need to account for the potential impact of adjuvant therapy selection on outcomes of those patients who relapse and receive additional systemic therapy. Secondly, despite its prospective design, our study included some variables with a significant amount of missing information, such as ECOG (45%), BRAF-mutation subtype (49%), and LDH (21%), which are prognostic variables with a potential impact on the reported outcomes. Nevertheless, we minimized the potential impact on our study result by conducting a sensitivity analysis excluding unknown BRAF-mutation subtype patients in a separate multivariable Cox model, which reported similar results as the initial multivariable analysis of the total population. Lastly, our study lacked the power of randomization, and therefore our results should be interpreted with caution. Until results from future randomized clinical trials become available, our study provides real-world evidence to potentially better guide clinicians in choosing first-line therapy in BRAF V600E/K-mutant melanoma patients.

## 5. Conclusions

Our nation-wide prospective study failed to establish any optimal systemic therapy sequencing patterns in advanced BRAF-mutant melanoma patients. Nevertheless, we provided evidence that Canadian medical oncologists were selecting appropriate treatment sequences based on patients’ clinical and tumour characteristics. Mature data from several phase III randomized controlled trials (NCT02631447 and NCT02224781) would be required to definitively determine optimal systemic therapy sequencing for the management of metastatic BRAF V600-mutant cutaneous melanoma patients.

## Figures and Tables

**Table 1:** Baseline study population characteristics (*n* = 186).

	Total	1L-IO Group	1L-TT Group	*p*-Value
	*n* (%)	*n* (%)	*n* (%)	
**Age**				
<65	93 (50)	43 (54)	50 (47)	0.374
≥65	93 (50)	36 (46)	57 (53)	
**Gender**				
Male	124 (67)	55 (70)	69 (54)	0.53
Female	62 (33)	24 (30)	38 (36)	
**ECOG**				
0–1	81 (44)	40 (91)	41 (72)	0.023
2 and above	20 (11)	4 (9)	16 (28)	
Missing	85 (45)	-	-	
**LDH**				
≥Median (280)	72 (39)	33 (52)	39 (48)	0.739
<Median	74 (40)	31 (48)	43 (52)	
Missing	40 (21)	-		
**Cancer Stage**				
Unresectable Stage III	9 (5)	4 (5)	5 (5)	1
Metastatic	177 (95)	75 (95)	102 (95)	
**Number of Metastatic Sites**				
>2	88 (47)	42 (53)	46 (43)	0.184
≤2	98 (53)	37 (47)	61 (57)	
**Baseline Brain Metastasis**				
Yes	46 (25)	17 (22)	29 (27)	0.397
No	140 (75)	62 (78)	78 (73)	
**BRAF-Mutant Types**				
V600E/K	89 (48)	35 (44)	54 (51)	0.326
V600D	5 (3)	1 (1)	4 (4)	
Unknown Subtypes	92 (49)	44 (55)	48 (45)	
**Received Palliative RT**				
Yes	108 (58)	46 (58)	62 (58)	1
No	78 (42)	33 (42)	45 (42)	
**Received Palliative Surgery**				
Yes	11 (6)	6 (8)	5 (5)	0.532
No	175 (94)	73 (92)	102 (95)	
**First Line Regimen**				
Anti-PD1	56	56 (71)	-	N/A
Anti-PD1 + CTLA-4	23	23 (29)	-	
BRAF + MEKi	100	-	100 (93)	
BRAFi	7	-	7 (7)	

**Table 2:** Characteristics of 2L therapy and timing of sequencing patterns (*n* = 186).

	1L-IO	1L-TT	*p*-Value
	*n* (%)	*n* (%)	
**Received 2L Therapy**			
Yes	35 (44)	56 (52)	0.302
No	44 (56)	51 (48)	
**Reason for 1L Treatment Discontinuation**			
Progression	29 (37)	50 (47)	N/A
Toxicity	17 (21)	13 (12)	
Treatment Completion	18 (23)	N/A	
Unknown	15 (19)	43 (41)	
**Progressed on 1L and Died before 2L Therapy**			
Yes	13 (45)	37 (74)	0.015
No	16 (55)	13 (26)	
**2L Therapy**			
Anti-PD1	0 (0)	41 (73)	N/A
Anti-PD1 + Anti-CTLA4	0 (0)	15 (27)	
BRAFi + MEKi	35 (100)	0 (0)	
**Reason for 2L Treatment Discontinuation**			
Progression	22 (62)	29 (52)	N/A
Toxicity	3 (9)	4 (7)	
Treatment Completion	7 (20)	N/A	
Unknown	3 (9)	23 (41)	
**Time on 1L Therapy**			
Median (Months)	5.3	4.2	0.791
25th and 75th Percentile (Months)	1.4, 12.3	2.6, 7.6	
**Time on 2L Therapy**			
Median (Months)	5.3	4.9	0.716
25th and 75th Percentile (Months)	2.3, 10.1	0.88, 18.8	
**Time from 1L Therapy Initiation Date to 2L Therapy Initiation Date**			
Median (Months)	5.5	6.2	0.338
25th and 75th Percentile (Months)	1.9, 11.7	3.6, 11.9	
**Time from 2L therapy Initiation Date to Death or Last Follow Up**			
Median (Months)	7.9	17.3	0.245
25th and 75th Percentile (Months)	4.2, 14.6	2.1, 32.9	

**Table 3:** Multivariable Cox analysis of study population.

Total Population (*n* = 186)			
	**Overall Survival**		
	**HR**	**95% CI**	***p*** **-Value**
**Number of Metastatic Sites > 2**	2.195	1.302–3.699	0.003
**Baseline Brain Metastasis**	1.833	1.073–3.130	0.027
**Baseline ECOG ≥ 2**	3.957	2.226–7.034	<0.001
**Sequencing Group** (1L-TT as Reference)	0.838	0.502–1.400	0.500
**BRAF V600E/K Mutant Population Only (*n* = 89)**			
	**Overall Survival**		
	**HR**	**95% CI**	***p*** **-Value**
**Number of Metastatic Sites > 2**	1.812	0.884–3.710	0.104
**Baseline Brain Metastasis**	1.903	0.944–3.836	0.072
**Baseline ECOG ≥ 2**	4.098	1.918–8.755	<0.001
**Sequencing Group** (1L-TT as Reference)	0.777	0.412–1.467	0.437

## Data Availability

Not applicable.
